# Do Different LED Colours Influence Sand Fly Collection by Light Trap in the Mediterranean?

**DOI:** 10.1155/2018/6432637

**Published:** 2018-06-27

**Authors:** Gabriella Gaglio, Ettore Napoli, Francesca Arfuso, Jessica Maria Abbate, Salvatore Giannetto, Emanuele Brianti

**Affiliations:** Dipartimento di Scienze Veterinarie, University of Messina, Messina, Italy

## Abstract

Light traps represent the most used attractive system to collect and monitor phlebotomine sand flies. Recent studies have suggested that light traps can be easily upgraded by the use of light-emitting diode (LED) with positive effects on trap design, weight, and battery life. However, scant data on the effect of different LED colours on the attractiveness to phlebotomine sand fly species are available in literature. In this study, the capture performances of light traps equipped with different LED colours on phlebotomine sand fly species indigenous in the Mediterranean area were evaluated. Phlebotomine sand fly collections were performed using a classical light trap (CLT), equipped with a traditional incandescent lamp, and five Laika 4.0 light traps supplied, each with LED of different colours and wavelengths: (i) white; (ii) red; (iii) green; (iv) blue; (v) UV. Light traps were set for three consecutive nights fortnightly from May to October 2017 and climate data recorded using a meteorological station. A total of 411 phlebotomine sand flies (191 males and 220 females), belonging to three different species, namely,* Phlebotomus perniciosus *(n= 298, 141 males and 157 females),* Sergentomyia minuta *(n=110, 48 males and 62 females), and* Phlebotomus neglectus *(n=3, 2 males and 1 females) were collected. Abundance of capture was influenced by colours of LED and time. The highest number of phlebotomine sand flies was captured on June (P<0.01) and by UV LED (P<0.01). As regard to species,* P. perniciosus *was mainly captured by UV LED on June (P<0.01). No effect of time (P>0.05) or LED colour (P>0.05) was recorded for* S. minuta *and* P. neglectus*. According to the results of the present study light trap equipped with UV LED can represent an effective tool for the capture of sand fly species in the Mediterranean area.

## 1. Introduction

Phlebotomine sand flies (Diptera: Psychodidae) are small nocturnal insects that act as vectors of various infectious and parasitic agents including canine and human* Leishmaniosis*. These insects play a crucial role in the epidemiology of relevant diseases being some of great veterinary and medical importance; their monitor and control are, therefore, of pivotal importance.

The system for trapping phlebotomine sand flies can be categorized into attractive (e.g., light traps and CO_2_) or passive (e.g., sticky traps) traps, and the different methods may influence the capture outcomes including specific attraction to sand fly species in a given area [1]. Although phlebotomine sand flies are nocturnal/crepuscular insects and their flight activity increases in relation to the decrease of the daily light intensity [2], they are contemporarily attracted by artificial light; thus, light traps are largely employed for the collection of these insects. Interestingly, insects are attracted by light of different colours and intensity relating to their retina structure and to the presence of photoreceptors. Honeybees, for instance, have three photoreceptors (i.e., ultraviolet, blue, and green); in butterfly the retinas have six or more photoreceptor classes [3]. It has been demonstrated that the eyes of the adult sand fly* Lutzomyia longipalpis* reacted maximally to light in the ultraviolet region (at 340 nm) with a secondary peak in the blue-green-yellow region at 520-546 nm [4].

Light trap methods have recently been improved by the use of light-emitting diode (LED) [5–8]. LED light traps have some advantages compared to traditional light trap models including the low electric consumption resulting in a longer battery life and in a longest lifetime of the lamp. The LED technology allows easily customizing the colour and the intensity of the light. Some authors showed that LED of different colours could have different power of attraction demonstrating, for instance, that* Phlebotomus papatasi* is more attracted by red LED [9], while* Nyssomyia whitmani* and* Lutzomyia longipalpis *seem to be more attracted by blue and green LED, respectively [10]. However, no data on the effect of different LED colours on other* Phlebotomus *species present in Mediterranean area are available in the literature. Therefore, the aim of the present study is to evaluate the capture performances of light traps equipped with different LED colours to phlebotomine sand fly species endemic in the Mediterranean area.

## 2. Materials and Methods

### 2.1. Study Area and Collection Procedures

The study was conducted from May to October 2017, which corresponds to the sand fly season in the study area [11], in the municipality of Messina, an area highly endemic for canine leishmaniosis where the presence of competent sand fly species has been previously reported [11, 12]. The traps were placed in a suburban area nearby the horse stables of the Department of Veterinary Sciences of the University of Messina (38°13′59′′N; 15°32′48.99′′E; 263 m a.s.l.).

**Figure 1:**
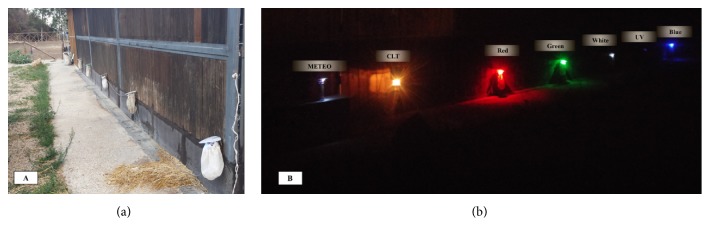
Classical light trap (CLT) and five Laika traps equipped with red, green, white, UV, and blue LEDs placed in the study area. (a) Diurnal vision; (b) nocturnal vision.

Sand fly collection was performed using both a classical light trap (named CLT), equipped with a traditional incandescent lamp (12V, 8W) and five Laika 4.0 light traps supplied, each with LED of different colours and wavelengths: (i) white/455 nm; (ii) red/620 nm; (iii) green/530 nm; (iv) blue/470 nm; (v) UV/395 nm. The six traps were set opposite to a stonewall at 50 cm above the ground [11] and at about 3 meters apart from each other (Figures 1(a) and 1(b)). Both CLT and LED traps were placed for three consecutive days twice a month (six days per month), from May to October 2017. Traps were switched-on before sunset (18:00) and left working for 13 hours (up to 7:00 a.m.). Net-bags of traps were collected and replaced after each day of collection. Temperature (°C), relative humidity (RH%), and wind intensity (WI) were recorded using a meteorological station placed in the same area of traps.

### 2.2. Sand Fly Identification

Phlebotomine sand flies collected were initially separated from other insects, differentiated by sex and stored in vials containing 70% ethanol. For species identification, the external genitalia of males and the head and posterior last tergites of females were dissected, cleared, and slide-mounted as described elsewhere [8]. Identification was performed using morphological keys [13].

### 2.3. Data Analysis

Due to the limited number of phlebotomine sand flies collected in each sampling session, the data were merged according to the month of capture. Two-way analysis of variance (ANOVA) was applied in order to evaluate the effect of time (i.e., month) and traps (i.e., CLT or LED traps) on abundance and sand fly species. When significant differences were found, Bonferroni's post hoc comparison was applied.

For the best represented species, Pearson's chi-square analysis was applied to evaluate statistically significant difference in the number of male and female specimens.

Statistical significant values were set for *P* values < 0.05. The statistical analyses were performed using the STATISTICA software package (STATISTICA 7 for Windows, Stat Software Inc., Tulsa, Oklahoma).

## 3. Results

**Figure 2:**
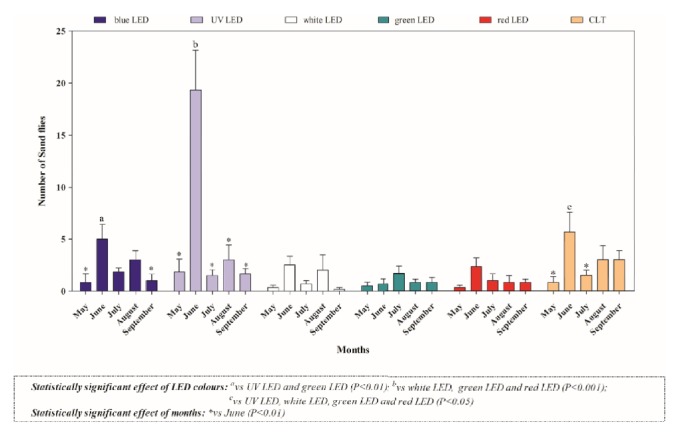
Total number of phlebotomine sand flies captured by classical light trap (CLT) and five Laika traps equipped with red, green, white UV, and blue LEDs.

**Figure 3:**
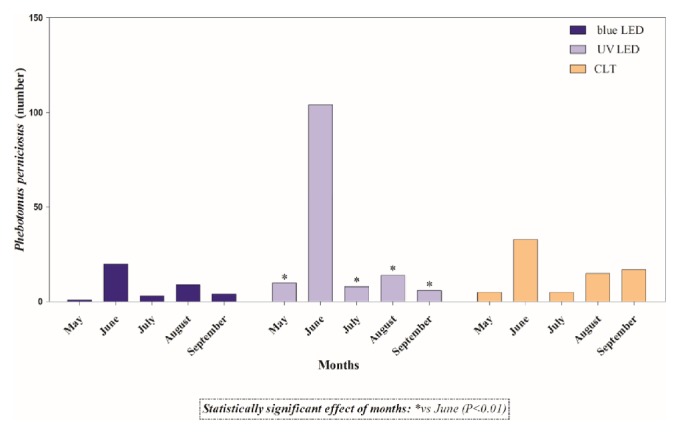
Total number of* Phlebotomus perniciosus* captured by classical light trap (CLT) and blue and UV Laika traps.

**Figure 4:**
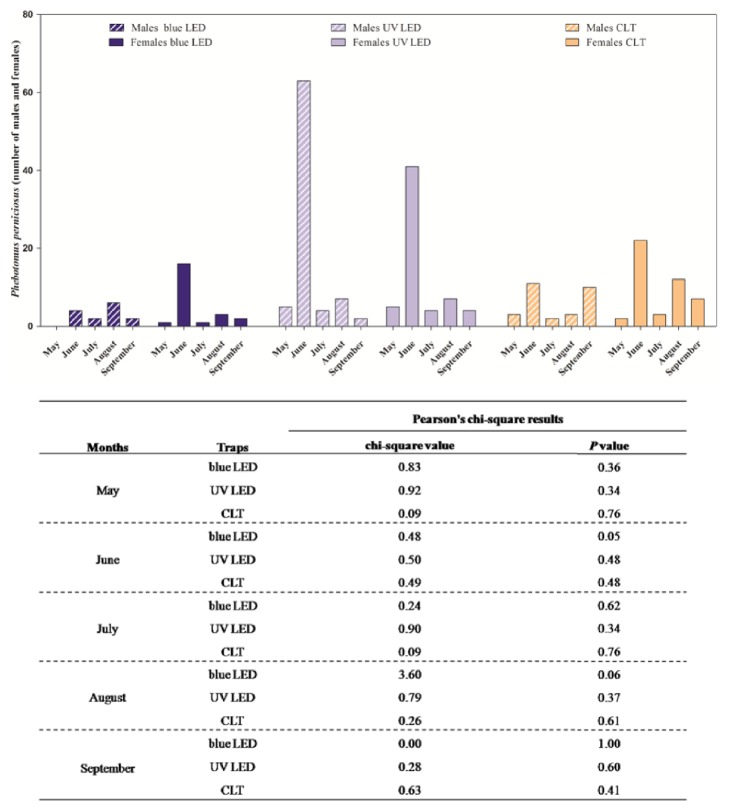
Number of males and females of* Phlebotomus perniciosus* captured by classical light trap (CLT) and blue and UV Laika trap along with results of Pearson's chi-square test.

A total of thirty-six sampling days were carried out throughout the study, but no phlebotomine sand flies were captured in the first four (May) and in the last six capture days (October). The number of captured phlebotomine sand flies along with environmental parameters recorded during the sampling days are summarized in Table 1. Overall, 411 specimens, belonging to three different species, namely,* Phlebotomus perniciosus *(n= 298, 141 males and 157 females),* Sergentomyia minuta *(n=110, 48 males and 62 females), and* Phlebotomus neglectus *(n=3, 2 males and 1 females) were collected (Table 2). A statistically significant effect of time (month) and trap model found that the highest number of specimens was collected in the month of June and by blue LED, UV LED, and CLT (Figure 2). Similarly, in regard to species,* P. perniciosus* was mainly captured in June (*P*<0.01) and by UV LED (Figure 3); no differences in the number of male and female specimens of* P. perniciosus *captured by the three more efficient traps (i.e., blue LED, UV LED, and CLT traps) were observed (Figure 4). Neither effect of sampling period nor of trap model was found on the number of* S. minuta *and* P. neglectus* captured during the study.

## 4. Discussions

The advent of LED technology has substantially improved light trap performances. Despite the fact that previous surveys have demonstrated how light trap using LED technologies can be valid alternative to classical models, the attractiveness of different colours LED has not been explored in deep, so far. The present study investigated the capture performances of light trap equipped with LED of different colour and wavelength on sand fly species endemic in the Mediterranean area. According to the herein results, it may be speculated that light trap equipped with UV LED of 395 nm has a higher attractiveness compared to other coloured LED traps. This is particularly evident for the species* P. perniciosus* which is one of the main vectors of leishmaniosis by* L. infantum* to both humans and dogs [14].

The species captured in the present survey are the same reported in other previous investigations performed in the same area [11, 12, 15], where* P. perniciosus* and* S. minuta* were the most abundant species. Though the well-known role of* P. perniciosus* is being competent vector of* L. infantum, *that of* S. minuta *is still unclear and needs further investigations. Recently, [16] reported the first molecular detection of* L. infantum* in* S. minuta *in southern Portugal and the first isolation of human blood as meal source in an engorged female of* S. minuta. *These findings opened a debate on the potential role of this species in the transmission of leishmaniosis and its involvement in the epidemiology deserves further researches including, but not limited to, protozoan isolation from engorged specimens as well as experimental transmission.

The limited presence of* P. neglectus* here observed is consistent with previous reports in southern Italy [11, 17] and justified by the higher abundance of this species in northern Italian regions which are featured by cooler climate [18, 19].

The attractiveness of different light technologies and colours could bias the estimation of sand fly population in a specific area. In fact, as demonstrated by a study that compared the attractiveness of different LED colours,* Phlebotomus papatasi* was attracted four times more by red LED compared to blue and green and twice with respect to incandescent lamp [9]. A study performed in Brazil demonstrated that Hoover Pugedo light traps equipped with green or blue LED or incandescent lamps showed the same attractiveness power for* Nyssomyia whitmani* and* Lutzomyia longipalpis* [10]. In another study, the CDC and Disney traps were, respectively, more efficient in the capture of* Lutzomyia ovallesi *and* Lutzomyia olmeca olmeca *compared to LED light trap [20]. In a previous study conducted by our group [8] the attractiveness of a Laika trap equipped with both white and UV LED was compared to CLT, no significant differences in the trapping performance of the two traps were recorded in that study. Here, the Laika model equipped with UV LED showed the best capture performances in general and to* P. perniciosus* in particular. These findings suggest that the eyes of* P. perniciosus* react maximally to light in the ultraviolet region as already observed for* Lutzomyia longipalpis* [4]. However, spectral sensitivities using electroretinograms need to be determined in order to clarify whether the greater attractiveness of the UV LED for this species is linked to a higher sensitivity of the eyes to wavelength of the ultraviolet region (395 nm) or to the low brightness emitted by this LED colour.

In addition to trapping methods, sand fly abundance and richness may be influenced considerably by other variables including animals' presence and environmental and climate variables [21]. In particular, the environmental factors have been used to predict and elucidate the distribution of diseases transmitted by vectors [22]. The climatic factors including temperature, humidity, wind, and rainfall could influence the distribution of vectors including sand fly species [22, 23]. A previous study conducted in the same area recorded two peaks of sand fly collection on August and September [11], in the present study instead only one peak was observed in the month of June. Moreover, phlebotomine sand flies were captured earlier in the here survey compared to the aforementioned study in which the first positive trapping session was observed in late June. In regard to this difference, it is interesting to notice that the summer season in which the present study was conducted was featured by very hot temperature especially in July (28.6 ± 3.2°C) and August (28.8 ± 5.0) and high humidity in September (69.3 ± 13.1%) and October (72.5 ± 15.6%) caused by intense rains. These particular climatic conditions may explain the decline in the number of sand flies during the hottest months and their complete absence in October.

In conclusion, this study showed how light trap equipped with UV LED of 395 nm has superior performances to other LED traps equipped with blue or green or white or red lamps for the capture of sand flies including important* Leishmania* vector species such as* P. perniciosus*. By virtue of their performances and technology, UV LED trap may represent a highly efficient alternative to CLT for the capture and monitoring of the sand fly species endemic in the Mediterranean.

## Figures and Tables

**Table 1:** Number of phlebotomine sand flies captured in the study along with environmental parameters recorded during the trapping days. Environmental parameters are provided as means of values recorded during each trapping day (i.e., from 6 p.m. to 7 a.m.).

**Month**	**Sampling day**	**Sand fly number**	**Temperature (**°**C)**	**Relative Humidity (**%**)**	**Wind Intensity (km/h)**
**May**	**15**	0	23	46	23
	**16**	0	24	64	18
	**17**	0	22	58	20
	**24**	0	23	63	24
	**25**	15	21	57	17
	**26**	13	25	49	12

**June**	**13**	41	24	57	21
	**14**	29	25	49	28
	**15**	46	24	61	26
	**27**	61	28	55	10
	**28**	12	29	50	13
	**30**	24	28	63	14

**July**	**11**	10	31	45	17
	**12**	10	33	36	19
	**13**	15	30	60	26
	**25**	3	25	73	25
	**26**	7	25	67	19
	**27**	4	27	54	22

**August**	**1**	7	33	30	19
	**2**	6	33	28	21
	**3**	3	34	27	27
	**29**	9	25	87	17
	**30**	39	23	84	11
	**31**	12	24	74	10

**September**	**11**	14	24	73	19
	**12**	8	22	84	20
	**13**	2	27	50	18
	**25**	8	22	62	15
	**26**	10	22	64	9
	**27**	3	21	82	9

**October**	**9**	0	17	88	8
	**10**	0	19	96	10
	**11**	0	26	68	13
	**16**	0	20	57	9
	**17**	0	20	65	9
	**18**	0	21	61	18

**Table 2:** Number and percentages of phlebotomine sand fly species captured by each trap model in the study.

**Traps**	**Species**	**Study period**				
		**May** n/total (%)	**June** n/total (%)	**July** n/total (%)	**August** n/total (%)	**September** n/total (%)
**blue LED**	*Phlebotomus perniciosus*	1/5 (20)	20/30 (66.7)	3/11 (27.3)	9/18 (50)	4/6 (66.7)
	*Sergentomyia minuta*	4/5 (80)	10/30 (33.3)	8/11 (72.7)	9/18 (50)	2/6 (33.3)
	*Phlebotomus neglectus*	0/5 (0)	0/30 (0)	0/11 (0)	0/18 (0)	0/6 (0)

**UV LED**	*Phlebotomus perniciosus*	10/11 (90.9)	104/116 (89.7)	8/9 (97.1)	14/18 (77.8)	6/10 (60)
	*Sergentomyia minuta*	1/11 (9.1)	12/116 (10.3)	1/9 (2.9)	4/18 (22.2)	4/10 (40)
	*Phlebotomus neglectus*	0/11 (0)	0/116 (0)	0/9 (0)	0/18 (0)	0/10 (0)

**white LED **	*Phlebotomus perniciosus*	2/2 (100)	9/15 (60)	0/4 (0)	9/12 (75)	1/1 (100)
	*Sergentomyia minuta*	0/2 (0)	5/15 (33.3)	4/4 (100)	3/12 (25)	0/1 (0)
	*Phlebotomus neglectus*	0/2 (0)	1/15 (6.7)	0/4 (0)	0/12 (0)	0/1 (0)

**green LED**	*Phlebotomus perniciosus*	1/3 (33.3)	2/4 (50)	3/10 (30)	0/5 (0)	4/5 (80)
	*Sergentomyia minuta*	2/3 (66.7)	2/4 (50)	7/10 (70)	5/5 (100)	0/5 (0)
	*Phlebotomus neglectus*	0/3 (0)	0/4 (0)	0/10 (0)	0/5 (0)	1/5 (20)

**red LED**	*Phlebotomus perniciosus*	1/2 (50)	10/14 (71.4)	0/6 (0)	0/5 (0)	2/5 (40)
	*Sergentomyia minuta*	1/2 (50)	4/14 (28.6)	6/6 (100)	5/5 (100)	2/5 (40)
	*Phlebotomus neglectus*	0/2 (0)	0/14 (0)	0/6 (0)	0/5 (0)	1/5 (20)

**CLT**	*Phlebotomus perniciosus*	5/5 (100)	33/34 (97.1)	5/9 (55.6)	15/18 (83.3)	17/18 (97.1)
	*Sergentomyia minuta*	0/5 (0)	1/34 (2.9)	4/9 (44.4)	3/18 (16.7)	1/18 (2.9)
	*Phlebotomus neglectus*	0/5 (0)	0/34 (0)	0/9 (0)	0/18 (0)	0/18 (0)

Legend: blue LED, UV LED, white LED, green LED, red LED= Laika traps equipped with blue, UV, white, green and red LEDs respectively; CLT = Classical light trap equipped with incandescent lamp.

## Data Availability

Data supporting the findings of this study is included within the article.
